# Acute stress activates basolateral amygdala neurons expressing corticotropin-releasing hormone receptor type 1 (CRHR1): Topographical distribution and projection-specific activation in male and female rats

**DOI:** 10.1016/j.ynstr.2024.100694

**Published:** 2024-11-15

**Authors:** Robert J. Aukema, Gavin N. Petrie, Samantha L. Baglot, Nicholas W. Gilpin, Matthew N. Hill

**Affiliations:** a Neuroscience Graduate Program, University of Calgary, Calgary, AB, T2N 4N1, Canada; b Hotchkiss Brain Institute, Cumming School of Medicine, University of Calgary, Calgary, AB, T2N 4N1, Canada; c Mathison Centre for Mental Health Research and Education, Cumming School of Medicine, University of Calgary, Calgary, AB, T2N 4N1, Canada; d Departments of Cell Biology & Anatomy and Psychiatry, Cumming School of Medicine, University of Calgary, Calgary, AB, T2N 4N1, Canada; e Department of Physiology, Louisiana State University, New Orleans, LA, 70112, USA

## Abstract

Although the basolateral amygdala (BLA) and corticotropin releasing hormone receptor type I (CRHR1) signaling are both central to the stress response, the spatial and circuit-specific distribution of CRHR1 have not been identified in the BLA at a high resolution. We used transgenic male and female CRHR1-Cre-tdTomato rats to topographically map the distribution of BLA^CRHR1^ neurons and identify whether they are activated by acute stress. Additionally, we used the BLA circuits projecting to the central amygdala (CeA) and nucleus accumbens (NAc) as a model to test circuit-specific expression of CRHR1 in the BLA. We established several key findings. First, CRHR1 had the strongest expression in the lateral amygdala and in caudal portions of the BLA. Second, acute restraint stress increased FOS expression of CRHR1 neurons, and stress-induced activation was particularly strong in medial subregions of the BLA. Third, stress significantly increased FOS expression on BLA-NAc, but not BLA-CeA projectors, and BLA-NAc activation was more robust in males than females. Finally, CRHR1 was expressed on a subset of BLA-CeA and BLA-NAc projection neurons. Collectively, this expands our understanding of BLA molecular- and circuit-specific activation patterns following acute stress.

## 1 Introduction

The basolateral amygdala (BLA) is a heterogenous brain region that is integral to processing emotional stimuli. It is activated by a wide range of psychological stressors (Aukema et al., 2024; Úbeda-Contreras et al., 2018) and can drive both anxiety-like behaviour and hypothalamic-pituitary-adrenal (HPA) responses to stress (Bhatnagar et al., 2004; Tye et al., 2011). These effects are likely mediated through projections to a wide range of limbic regions such as the prelimbic cortex, ventral hippocampus, nucleus accumbens, bed nucleus of the stria terminalis, and central amygdala (Sah et al., 2003). Indeed, there are multiple lines of evidence that individual BLA projection neuron populations can drive changes in anxiety-like behaviour (Janak and Tye, 2015). However, the BLA also responds to rewarding stimuli (Beyeler et al., 2018; Shabel and Janak, 2009; Zhang et al., 2020), and individual projection neuron populations are known to drive a wide range of different behavioural responses apart from anxiety-like behaviour (Beyeler et al., 2018; Janak and Tye, 2015; Kim et al., 2016). Further, within single projection neuron populations, varied and even opposing influences on behaviour can be observed (Bagot et al., 2015; Beyeler et al., 2018; Birnie et al., 2023; Folkes et al., 2020; Shen et al., 2019; Stuber et al., 2011). This suggests strong heterogeneity even within single projection populations and may be reflected in differences in molecular identity. Thus, alongside circuit identity, identification and characterization of molecular markers for stress-activated cells may be an important complementary approach to classifying projection neuron populations. Yet, this remains a critical gap in our understanding of stress-activated neurons in the BLA (McCullough et al., 2016a, McCullough et al., 2016b).

Corticotropin-releasing hormone (CRH) is a central regulator of the stress response. The CRH receptor Type I (CRHR1) is widely expressed across limbic regions (Weera et al., 2022), and global pharmacological inhibition or genetic deletion of CRHR1 dramatically reduces both anxiety-like behaviour and HPA responses to stress (Habib et al., 2000; Müller et al., 2003; Smith et al., 1998). More specifically, CRHR1 is moderately expressed in the BLA, predominantly in glutamatergic projection neurons (Agoglia et al., 2020; Chen et al., 2000; Van Pett et al., 2000), and CRH is released into the amygdala during acute stress in rodents such as restraint stress (Merlo Pich et al., 1995). Functionally, CRH injection directly into the BLA leads to activation of CaMKiia projection neurons (Rostkowski et al., 2013), enhances memory consolidation (Liang and Lee, 1988; Roozendaal et al., 2002), drives anxiety-like behaviour, and amplifies the HPA response to stress (Gray et al., 2015). Thus, release of CRH and activation of CRHR1 during stress likely contributes to multiple aspects of the stress response, perhaps through activation of discrete BLA circuits. As a result, CRHR1 may represent a unique molecular phenotype of stress-responsive cells in the BLA.

Precise anatomical and circuit mapping of CRHR1 neurons has been difficult due to a lack of reliable anti-CRHR1 antibodies and relatively low protein expression levels in the BLA (Refojo et al., 2011; Weera et al., 2022). It thus remains poorly understood which specific subregions and projection neuron populations of the BLA that CRHR1 is expressed within. Given that distinct subregions of the BLA appear differentially responsive to aversive and rewarding stimuli (Aukema et al., 2024; Hale et al., 2006; Kim et al., 2016) and house distinct projection neuron populations (Beyeler et al., 2018; Huang et al., 2021; McGarry and Carter, 2017; Reppucci and Petrovich, 2016), identifying the topographical distribution of CRHR1 in the BLA will be informative for both establishing which discrete BLA projection circuits CRHR1 are expressed within as well as what behavioural and physiological processes this population may contribute to.

Recently, Weera and colleages (2022) have generated a CRHR1-Cre-tdTomato transgenic rat, allowing both the visualization of CRHR1 distribution as well as manipulation of these neurons through genetic tools such as cre-dependent viral constructs (Weera et al., 2022, 2023). We therefore used this transgenic line to anatomically map CRHR1 expression throughout the BLA and to establish if BLA^CRHR1^ neurons are activated by stress. We then used BLA projection populations targeting the nucleus accumbens (NAc) and central amygdala (CeA) as a model for interrogating circuit-specific expression of CRHR1. We selected these projection populations as they are anatomically distinct (Beyeler et al., 2018) and known to drive both appetitive and aversive behaviours (Beyeler et al., 2018; Birnie et al., 2023; Folkes et al., 2020; Kim et al., 2017; Shen et al., 2019; Stuber et al., 2011; Tye et al., 2011).

Here, we anatomically describe the topographical and circuit-specific distribution of BLA^CRHR1^ neurons. Importantly, we investigate whether this population is activated by acute stress. Together, this combines circuit- and molecular-specific approaches to provide insight into discrete BLA projection neuron populations activated by acute stress in adult male and female rats.

## 2 Materials and methods

### 2.1 Animals

All animal protocols were approved by the University of Calgary Animal Care Committee and followed guidelines from the Canadian Council on Animal Care. Adult male and female CRHR1-Cre-tdTomato rats on a Wistar background (Weera et al., 2022) were bred within the animal facility (10–13 weeks at time of testing). Rats were maintained under a 12h light-dark cycle (lights on at 8am) with food and water available *ad libitum* and pair-housed for the duration of the experiment. Cage-mates were always in identical treatment groups and underwent all aspects of experimentation at the same time. No more than 3 animals (and 2 of the same sex) from each litter were used per condition, such that a maximum of 30% of animals in each group were ever from the same litter.

### 2.2 Genotyping

Rats were genotyped for the presence of the iCre transgene by extracting DNA from ear notch samples using the Quantabio Extracta DNA prep according to manufacturer's instructions, followed by PCR amplification with Kapa2G Fast Genotyping Mix using the following primers: iCre-F (AGATGCCAGGACATCAGGAACCTG), iCre-R (ATCAGCCACACCAGACACAGAGATC), rROSA26-F (CTTCAGCCACATGGTGGGTC) and rROSA26-R (TTGGCTAACTTACCAGTTATGCTACCT). Rat samples containing the iCre transgene resulted in a specific PCR product of 236 bp whereas the control ROSA26 product appeared at 826 bp in all samples. Only animals expressing iCre were included for experimentation.

### 2.3 Stereotaxic surgery

Rats were maintained under isoflurane anesthesia and analgesic treatment (meloxicam (2 mg/kg, subcutaneously)) in a stereotaxic apparatus during surgery. A glass capillary containing Alexa-conjugated Cholera Toxin Subunit B (CTB-488; Invitrogen; 0.2% in 0.04M PBS) was lowered into the brain and pressure injected using a NanoInject II apparatus (Drummond Scientific). All injections targeted the right hemisphere only. Coordinates are relative to bregma and the surface of the skull; for NAc: AP +1.8 mm, ML -1.4, DV -7.2; for CeA: AP -2.1 mm, ML -4.0, DV -8.2. A total of 322 nl of CTB-488 was delivered to the NAc (7 × 46 nl boluses over 6min) and 230 nl to the CeA (5 × 46 nl boluses over 4min). Following injection of the last bolus, the glass capillary remained in place for an additional 10min to allow diffusion of CTB-488. Animals were allowed to recover for a minimum of 1 week before any handling, habituation, or testing began.

### 2.4 Restraint stress

On experiment day, animals were carted to an adjacent experimental room from the colony room and placed into clear Plexiglas restraint tubes for 30min. Animals were then returned to their home cage with their cage-mate and remained in the testing room until 90min following stress-onset, where they were rapidly anesthetized with an overdose of sodium pentobarbital (*i.p.*) and transported to a separate room for perfusion and brain collection. Control animals remained in the colony room until time of sacrifice, when they were immediately anesthetized, perfused, and brains collected.

### 2.5 Tissue collection

Brains were collected and processed identically for all experiments. For perfusion, animals were anesthetized with an overdose of sodium pentobarbital and transcardially perfused with 0.9% saline (∼60 mL per rat, 30 mL/min) followed by 3.8% paraformaldehyde in 0.01M PBS (∼120 mL per rat, 30 mL/min). Following perfusion, brains were removed and immersed in 3.8% paraformaldehyde in 0.01 M PBS overnight before being switched to a 20% sucrose solution in PBS for 48–72 h, and then transferred to a 30% sucrose solution in PBS for cryoprotection. Coronal sections of 40um were cut in four series’ on a Leica SM 2010R sliding microtome and collected in antifreeze (30% wt/vol sucrose, 1% wt/vol polyvinylpyrrolidone-40, 30% vol/vol ethylene glycol, 0.0065% wt/vol sodium azide, in PBS (adapted from (Butler et al., 2012)) and stored at −20 C until processing.

### 2.6 Immunohistochemistry

Free-floating sections of the BLA were rinsed 3 × 10min in PBS, followed by 3 × 10min in PBS + Triton X-100 (0.1%). Sections were then blocked for 1h at room temperature under gentle agitation in 5% normal donkey serum in PBS and incubated in primary antibody at 4 °C in antibody blocking solution (0.1% vol/vol Triton X-100, 0.1% wt/vol BSA, 0.05% wt/vol sodium azide, 0.04% wt/vol sodium EDTA in PBS). The primary antibodies used were anti-cFos antibody raised in rabbit (cFos, Cell Signaling Technology #2250s, 1:400, 24h incubation) or anti-RFP antibody raised in goat (RFP, Rockland #200-101-379. 1:1000, 48h incubation). Following incubation, sections were washed 3 × 10min in PBS + Triton X-100 (0.1%) and incubated for 2h at room temperature with secondary antibody in antibody solution. The secondary antibodies used were donkey anti-rabbit AlexaFluor 647-conjugated secondary antibody (Alexa-647, #711-605-152, Jackson ImmunoResearch, 1:125) or Cy3 donkey anti-goat (Cy3, Jackson #705-165-147, 1:500). Finally, sections were rinsed 3 × 10min in PBS + Triton X-100 (0.1%) and 2 × 10min in PBS, mounted onto charged slides, and cover-slipped using Fluoroshield with DAPI mounting medium (Sigma Aldrich).

### 2.7 Imaging

Exposure settings were identical between all images within the same experiment, and the experimenter was blinded to sex and condition of the animal during acquisition and analyses.

*For topographical mapping of entire coronal sections:* after immunohistochemistry for RFP, tilescan images of the BLA from both hemispheres were collected between AP -2.12 to AP -3.60 (Paxinos & Watson atlas) from each animal using an Olympus VS110 Slidescanner with a 20X (0.75 NA air) objective. Only images with tissue undamaged throughout processing, sectioning, and immunohistochemistry were included (number of animals and slices per group: males = 5(36); females = 5(34).

*For histological verification of CTB-488 injections:* Images of the CeA or NAc were acquired using an Olympus VS120 slidescanner using a 10X/0.4 NA air objective. Location of maximal expression of CTB-488 was plotted onto coronal images adapted from an atlas (Swanson, 2004).

*For colocalization of CTB-488, FOS, and tdTomato:* After immunohistochemistry for FOS, images of the medial or lateral BLA (AP -2.76 to AP -3.48; Fig. 2B) from both hemispheres were collected using a Leica TCS SPE II confocal microscope using a 20X/0.55 NA HC PL FLUOTAR objective (Leica 5065190). cFos, CTB-488, and tdTomato signal were acquired independently and exported to ImageJ for quantification.

### 2.8 Topographical mapping and quantification of CRHR1+ cells

**Fig. 1:**
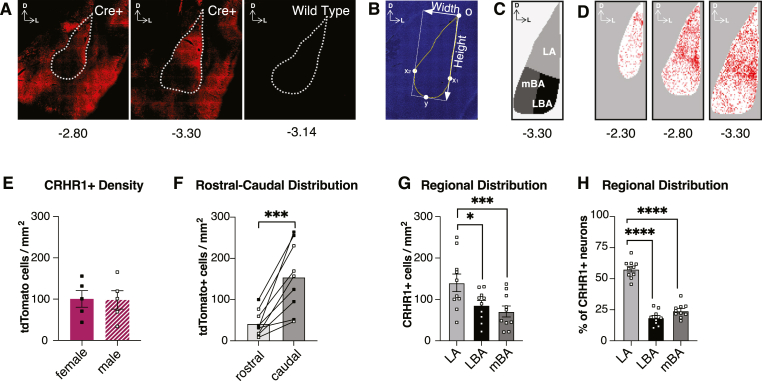
Topographical distribution of CRHR1 neurons in the basolateral amygdala. **(A)** Representative image of CRHR1-tdTomato expression following immunohistochemistry for RFP in transgenic CRHR1-tdtomato (left, middle) and wild type rats (right). Distance of coronal section from bregma; dashed lines delineate boundary of BLA. **(B)** Representative image of BLA depicting normalization procedure. Curved dashed line indicates boundary of BLA as determined by DAPI staining; ‘o’ indicates origin of reference frame; x_1_ indicates most lateral point of BLA; x_2_ indicates most medial point of the BLA; y indicates most ventral point of the BLA; white arrows indicate measured width and height of the BLA; **(C)** BLA subdivisions at AP -3.30 following normalization procedure: lateral amygdala (LA), lateral basal amygdala (LBA), and medial basal amygdala (mBA). **(D)** Heatmaps representing density of normalized CRHR1+ expression in 25um x 25um bins at AP -3.30. Darker colour represents higher density. **(E)** There were no significant differences in BLA^CRHR1^ expression between males and females (t(8) = 0.08763, *p* = 0.9323; n = 5 per sex). **(F)** There was significantly greater CRHR1+ density in caudal sections of the BLA (t(8) = 5.164, p = 0.0009). Data includes both sexes (n = 4 males; n = 4 females). Rostral sections include AP -2.12, −2.30, −2.56; caudal sections include AP -3.14, −3.30, −3.60. **(G)** There was significantly greater CRHR1 density in the LA than other BLA subregions (F(1.642,14.78 = 16.19, *p* = 0.0003; Tukey's post-hoc comparisons indicate LA vs. LBA: *p* = 0.0158; LA vs. mBA: *p* = 0.0008). Data include both sexes (n = 5 males; n = 5 females). **(H)** There was a significantly greater proportion of total CRHR1+ cells in the LA than other subregions (F(1.777, 16) = 76.01, *p* < 0.0001; Tukey's post-hoc comparisons indicate LA vs. LBA: *p* < 0.0001; LA vs. mBA: *p* < 0.0001). Data include both sexes (n = 5 males; n = 5 females). Data in 1E were analyzed using an unpaired *t*-test; data in 1F were analyzed using a paired *t*-test; data in 1G-H were analyzed using a RM one-way ANOVA. CRHR1-tdTomato+ neurons were quantified across the entire BLA (AP -2.12 to AP -3.60) and normalized according to average BLA dimensions at each AP position and number of images. Error bands represent mean+/-SEM. ∗*p* < 0.05, ∗∗∗*p* < 0.001; ∗∗∗∗*p* < 0.0001. (For interpretation of the references to colour in this figure legend, the reader is referred to the Web version of this article.)

Topographical mapping and quantification of CRHR1 cells was performed in both hemispheres. The experimenter remained blinded to the conditions of each animal during plotting and counting. Analyses were largely guided by work from Beyeler et al. (2018) and performed similar to our previous work (Aukema et al., 2024). In brief, we used Imaris Cell Imaging Software (Oxford Instruments) to semi-automatically detect RFP+ (CRHR1+) cells using the spot detection function. DAPI staining (Sigma-Aldrich) was used to visually identify the shape of the BLA based on contours provided by the surrounding fiber tracts. We then normalized all RFP + neurons of each section to a standardized shape of the BLA using a custom MATLAB script, by localizing the position of each RFP + cell to the most dorsal point of the BLA and the average dimensions of the BLA (Fig. 1B). This allowed us to accurately visualize average density gradients across multiple tissue sections from multiple animals. We then subdivided the BLA into three subregions approximated from the Paxinos & Watson atlas (Paxinos and Watson, 2007) to quantify average normalized CRHR1+ density per subregion. Further details on the normalization procedure are described in Supplementary Methods.

### 2.9 CTB-488, FOS, and tdTomato colocalization

tdTomato quantification was performed without any immunohistochemical amplification of RFP and only animals with greater than 50 tdTomato + cells identified across all sections were included. Any animals where CTB-488 injection occurred outside of the NAc or CeA, or was absent, and any images with BLA tissue damaged during brain dissection, was excluded. The same experimenter performed all manual and automatic counting and was blinded to the conditions and sex of each animal during image analysis and quantification. Number of cells expressing FOS were counted automatically in ImageJ based on minimum size and threshold parameters that remained identical across quantification for each experiment. Number of cells expressing tdTomato and CTB-488 were counted manually. Double or triple labelling of CTB-488, FOS, and tdTomato were determined as a FOS + signal bound by expression of CTB-488 (Jin and Maren, 2015).

### 2.10 Statistical analyses

Details for each statistical test are listed in each figure legend. GraphPad Prism 9 software was used for all statistical analyses. To compare the means of 2 unrelated groups on a single measure, we performed an unpaired *t*-test. To compare within-subject differences at 3+ measures, we performed a RM one-way ANOVA with the Geisser-Greenhouse correction followed by Tukey's post-hoc test. To compare within-subject differences between sexes, we performed a 2Way RM ANOVA followed by Sidak's post-hoc test. To compare differences with both sex and condition as a factor, we performed a 2Way ANOVA followed by Fisher's LSD. Statistical significance was defined as *p* < 0.05. Post-hoc tests were only performed when an interaction was detected (*p* < 0.10).

## 3 Results

### 3.1 Topographical distribution of CRHR1 neurons in the basolateral amygdala

We used adult male and female transgenic Wistar CRHR1-Cre-tdTomato rats (n = 5 male; n = 5 female) to map the expression pattern of CRHR1 in the BLA. Animals were first screened for the presence of transgenic iCre DNA using PCR. tdTomato was used as a marker for CRHR1 expression as both proteins are expressed as a single polypeptide (iCre-2A-tdTomato) that is cleaved at the 2A site (Weera et al., 2022). Co-expression of *Crhr1* and *iCre* has previously been validated in the BLA using this transgenic rat line (Weera et al., 2022). Importantly, these animals display behavioural and physiological phenotypes consistent with wild-type Wistar rats (Weera et al., 2022), including measures of nociception (Itoga et al., 2016), avoidance behaviours (Albrechet-Souza et al., 2020), and electrophysiological properties of amygdala neurons (Herman and Roberto, 2016).

tdTomato expression was strongly observed in the dorsal BLA following anti-RFP immunohistochemistry (Fig. 1A). To directly compare density gradients across animals we established a standardized BLA shape to normalize each individual data point to. We first measured the dimensions of the BLA of each image (Fig. 1B). There were no significant differences between sex in average BLA width (Supplementary Figure 1A), height (Supplementary Figure 1B) or triangular area (Supplementary Figure 1C) at each rostral-caudal plane from AP -2.12 to AP -3.60. We therefore used coordinates from both sexes to generate a standardized template for each rostral-caudal plane guided by the Paxinos & Watson atlas (2007) that subdivided the BLA into three distinct divisions (similar to (Aukema et al., 2024): lateral amygdala (LA), medial basal amygdala (mBA), and lateral basal amygdala (LBA; Fig. 1C). We then mapped the location of CRHR1-tdTomato+ cells relative to the most dorsal point of the BLA and normalized each coordinate to the average width and height of the BLA at each AP plane, using these normalized coordinates to express density gradients of CRHR1+ throughout the BLA (Fig. 1D) and quantify differences in CRHR1 density. There were no differences between males and females in average density of CRHR1+ cells across all sections of the BLA (Fig. 1E). We thus collapsed data from both sexes in further topographical analyses. In the rostral-caudal axis, there was significantly greater CRHR1 density in caudal sections of the BLA (Fig. 1F). In the dorsal-ventral and medial-lateral axis, there was significantly greater CRHR1+ density (Fig. 1G) and percentage of total quantified CRHR1+ cells (Fig. 1H) in the LA compared to both the mBA and LBA. Visually, the densest expression pattern was observed in the ventral aspects of the LA, and we therefore focused further investigation to this subregion. Collectively, these data demonstrate that the strongest expression of CRHR1+ neurons in both sexes is in the ventral LA subregion, particularly in caudal sections.

### 3.2 Stress robustly activates medial basolateral amygdala neurons expressing CRHR1

**Fig. 2:**
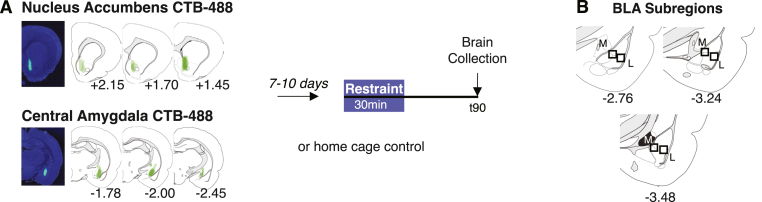
Stress robustly activates BLA neurons in the medial basal amygdala expressing CRHR1 **(A)** Experimental overview. Male and female CRHR1-tdTomato rats were injected with CTB-488 into either the nucleus accumbens (top) or central amygdala (bottom). 7–10 days later animals were exposed to 30min restraint stress (or home cage control) and perfused 90min later for imaging. Representative images depict area of maximal CTB-488 expression at the injection site. **(B)** Representative regions-of-interest where images were collected, according to the Swanson atlas (third edition; 2004); M = medial region; L = lateral region.

We next investigated whether CRHR1+ neurons in the BLA are activated by acute psychological stress. Adult male and female CRHR1-iCre-tdTomato rats were surgically injected with the retrograde tracer CTB-488 into the right NAc or CeA, and 7–10 days later tissue was collected immediately upon removal from the colony (naïve condition) or 90min following onset of 30min restraint stress (stress condition; Fig. 2A). We then quantified expression of CRHR1-tdTomato+, FOS+, and CTB-488+ neurons from images collected in the junction between the ventral LA and the dorsal basal amygdala, and including both lateral and medial subregions of the BLA (Fig. 2B). We specifically chose these two BLA regions of interest, as they both visually expressed the greatest density of CRHR1 neurons within the BLA (Fig. 1A–D) and differentially express BLA-NAc and BLA-CeA projection populations (Beyeler et al., 2018). Images were quantified from both left and right hemispheres.

**Fig. 3:**
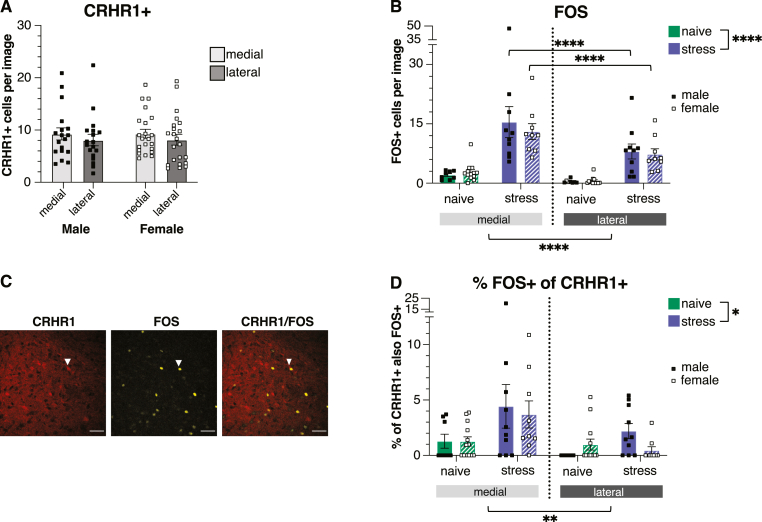
Stress robustly activates medial basal amygdala neurons expressing CRHR1 **(A)** There were no significant differences in density of CRHR1+ cells between sex (F(1,38) = 0.0015, *p* = 0.9698), BLA subregion (F(1,38) = 2.410, *p* = 0.1288), or an interaction of the two (F(1,38) = 0.0003, *p* = 0.9852). **(B)** There was significantly greater BLA FOS in animals exposed to stress (F(1,36) = 31.98, *p* < 0.0001) and in the medial region (F(1,36) = 48.40, *p* < 0.0001), but no main effect of sex (F(1,36) = 0.1075, *p* = 0.7449). There was also a significant interaction between condition and BLA subregion (F(1,36) = 14.55, *p* = 0.0005), with significantly greater FOS expression in the stress condition in the medial region in both males (*p* < 0.0001) and females (*p* = 0.0017) compared to the lateral region. **(C)** Representative mBA image of CRHR1-tdTomato fluorescence (top), FOS (middle), or colocalization of tdTomato and FOS (bottom). White arrows indicate colocalized cells. Scale bar, 50um. **(D)** Stress significantly increased the percentage of CRHR1+ cells expressing FOS (F(1,36) = 6.980, *p* = 0.0121). There was also a significantly greater percentage of CRHR1+/FOS+ in the medial region (F(1,36) = 1.822, *p* = 0.0069). There was no main effect of sex (F(1,36) = 0.3155, *p* = 0.5778) and no significant interactions. Data in 2A were analyzed using a 2Way RM ANOVA; data in 2B and D were analyzed using a three-way ANOVA with data matched by subregion (naive: n = 8 males, 13 females; stress: n = 10 males, 9 females). Error bands represent mean+/-SEM. ∗*p* < 0.05, ∗∗*p* < 0.01, ∗∗∗*p* < 0.001, ∗∗∗∗*p* < 0.0001.

We first replicated our above finding of CRHR1 expression in these regions. There were no differences in CRHR1 expression between sexes, hemispheres or between the lateral and medial BLA (Fig. 3A; Supplementary Figure 2A). There were also no differences in CRHR1 expression between conditions (Supplementary Figure 2B), suggesting stable expression. We next tested if stress differentially increases FOS expression in these subregions. As expected, there was significantly greater FOS expression in the stress condition (Fig. 3B). Consistent with our previous work (Aukema et al., 2024), we also observed a significant interaction, such that there was significantly greater FOS expression in the medial BLA than the lateral BLA following stress (Fig. 3B). There were no significant differences in stress-induced FOS expression between sexes or hemisphere (Supplementary Figure 2C), although there was a trend towards increased stress-induced FOS in the right hemisphere. Together, this suggests that although CRHR1+ neurons are similarly expressed in both the medial and lateral BLA, medial regions are particularly responsive to acute stress.

Next, we investigated whether stress activates CRHR1 neurons in the BLA by quantifying the percentage of CRHR1+ neurons colocalized with FOS expression following stress or the naïve condition (Fig. 3C). Stress significantly increased the percentage of CRHR1 neurons expressing FOS, and there was a significantly greater percentage of colocalized neurons in the medial BLA than the lateral BLA (Fig. 3D). There were no significant differences between sex. Collectively, our findings indicate that stress activates CRHR1+ neurons in the BLA, particularly CRHR1 cells located medially.

### 3.3 Basolateral amygdala projection populations are anatomically distinct and differentially activated by stress

**Fig. 4:**
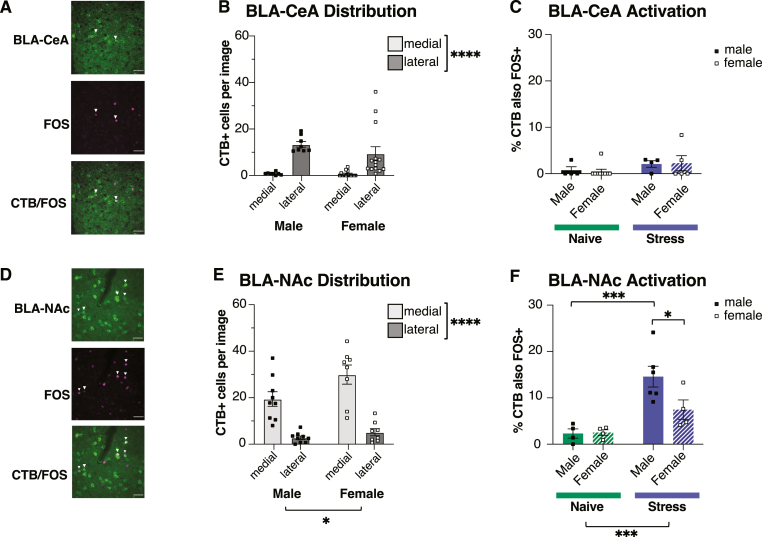
BLA projection populations are anatomically distinct and differentially activated by stress **(A)** Representative image of CTB-488 (top), FOS (middle), or colocalization of CTB-488 and FOS (bottom) following CTB injection into the CeA. White arrows indicate colocalized cells. Scale bar, 50um. **(B)** There were significantly more CeA projectors in the lateral than the medial region (F(1,20) = 34.28, *p* < 0.0001). There were no significant differences between sex (F(1,20) = 0.8446, *p* = 0.3690) and no interaction (F(1,20) = 1.142, *p* = 0.2980). **(C)** There were no significant differences between stress condition (F(1,18) = 2.629, *p* = 0.1223) or sex (F(1,18) = 0.0035, *p* = 0.9536) in percentage of BLA-CeA projectors also expressing FOS, and no interaction (F(1,18) = 0.04602, *p* = 0.8325). Data were analyzed from both lateral and medial BLA subregions. **(D)** Representative mBA image of CTB-488 (top), FOS (middle), or colocalization of CTB-488 and FOS (bottom) following CTB injection into the NAc. White arrows indicate colocalized cells. Scale bar, 50um. **(E)** There were significantly more NAc projectors in the medial than the lateral region (F(1,15) = 78.59, *p* < 0.0001) and in females compared to males (F(1,15) = 4.648, *p* = 0.0477). There was no significant interaction between sex and region (F(1,15) = 3.041, *p* = 0.1016) **(F)** There were significantly more BLA-NAc projectors that also expressed FOS in the stress condition (F(1,14) = 20.54, *p* = 0.0005). There was no significant effect of sex (F(1,14) = 3.332, *p* = 0.0894). There was a trending interaction between sex and region (F(1,14) = 3.749, *p* = 0.0733), and Fisher's LSD revealed significantly greater colocalization in males vs females in the stress condition (*p* = 0.0145). Data were analyzed from both the lateral and medial BLA subregions. Cells were quantified from images collected in the right hemisphere only. Data were analyzed using a 2Way ANOVA for both BLA-CeA (naïve: n = 4 males, 9 females; stress: n = 4 males, 5 females) and BLA-NAc (naïve: n = 4 males, 4 females; stress: n = 5 males, 4 females). Error bands represent mean+/-SEM. ∗*p* < 0.05, ∗∗*p* < 0.01, ∗∗∗*p* < 0.001, ∗∗∗∗*p* < 0.0001.

We next investigated whether discrete BLA projection neuron populations targeting the CeA or NAc are activated by acute stress, and whether this differs between sex. Using CTB-488 to define BLA-CeA or BLA-NAc projection neurons, we quantified colocalization of each projection population with FOS (Fig. 4A–D). Similar to others’ findings (Brog et al., 1993; Huang et al., 2019), CTB expression was largely restricted to the ipsilateral BLA as the injection site (data not shown); therefore, quantification and imaging was performed only in the right hemisphere for all projection-specific analyses and included a medial and lateral region-of-interest centred on the junction between the ventral LA and the basal amygdala as above. There were significantly more BLA-CeA projection neurons in the lateral region, and this did not differ by sex (Fig. 4B). We then quantified total percentage of BLA-CeA projection neurons also expressing FOS. Surprisingly, stress did not increase FOS expression on BLA-CeA projectors within the ventral LA in either sex (Fig. 4C). In contrast to the lateral bias of BLA-CeA projectors, there were more BLA-NAc projectors in the medial region; additionally, there were more BLA-NAc projectors in females than males (Fig. 4E). Unlike BLA-CeA projectors, stress increased the percentage of BLA-NAc neurons expressing FOS (Fig. 4F). Although there were no significant differences between sex, there was a trend towards a significant interaction between sex and region; post-hoc comparisons revealed that a greater proportion of BLA-NAc projectors were activated by stress in males (Fig. 4F). However, there were no differences between sex in average number per slice of BLA-NAc projectors also expressing FOS (Supplementary Figure 2D), suggesting a similar overall number of activated BLA-NAc projectors in both sexes, and these differences may instead be a result of a larger overall BLA-NAc population in females. Collectively, this demonstrates that BLA-NAc and BLA-CeA projection neurons are topographically and functionally distinct populations, with BLA-NAc projectors predominantly expressed medially and BLA-CeA projectors predominantly expressed laterally. Functionally, BLA-NAc projectors, but not BLA-CeA projectors within the ventral LA, are activated by stress. Further, the BLA-NAc projection population may be denser in females than it is in males.

### 3.4 CRHR1 is expressed proportionally more on CeA projectors than NAc projectors

**Fig. 5:**
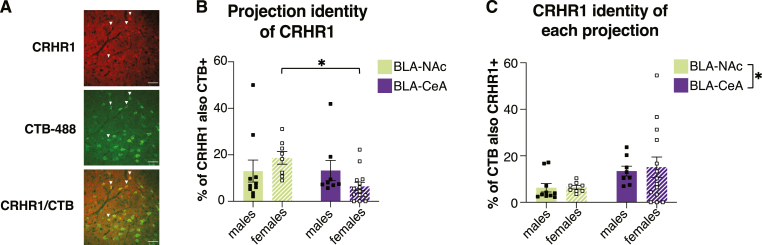
CRHR1 is expressed proportionally more on CeA projectors than NAc projectors **(A)** Representative mBA image of CRHR1 (top), CTB-488 (middle), or colocalization of CTB-488 and CRHR1 (bottom). White arrows indicate colocalized cells. Scale bar, 50um. **(B)** There were no main effects on proportion of CRHR1+ cells also expressing CTB by sex (F(1,36) = 0.0272, *p* = 0.8699) or projection (F(1,36) = 3.056, *p* = 0.0890), although there was a trending between the two (F(1,36) = 3.308, *p* = 0.0773). Fisher's LSD revealed that there were significantly more CRHR1 cells that projected to the NAc than the CeA in females only (*p* = 0.0130). **(C)** There was a significantly greater percentage of BLA-CeA projectors that expressed CRHR1 than BLA-NAc projectors (F(1,36) = 5.197, *p* = 0.0287). There were no main effects of sex (F(1,36) = 0.0739, *p* = 0.7872) or an interaction between the two (F(1,36) = 0.0466, *p* = 0.8302). Data were analyzed using a 2Way ANOVA for both BLA-NAc (n = 10 males, 8 females) and BLA-CeA (n = 8 males, 14 females). Error bands represent mean+/-SEM. ∗*p* < 0.05, ∗∗*p* < 0.01, ∗∗∗*p* < 0.001, ∗∗∗∗*p* < 0.0001.

Finally, we investigated whether CRHR1 is expressed on BLA-CeA and BLA-NAc projection neuron populations (Fig. 5A). There were no significant differences between sex, condition, or projection population, although post-hoc comparisons revealed a significantly greater proportion of all quantified CRHR1 neurons projecting to the NAc than the CeA in females only (Fig. 5B). On average in males, a similar proportion of CRHR1 neurons projected to the CeA (13.25%) as the NAc (13.00%); in females, a significantly greater proportion of CRHR1 neurons projected to the NAc (18.69%) than the CeA (6.42%).

Likewise, we investigated whether there were differences in the proportion of BLA-CeA and BLA-NAc projectors expressing CRHR1. There was a significantly greater percentage of BLA-CeA projectors expressing CRHR1 than BLA-NAc projectors, but no differences between sex (Fig. 5C). On average, 13.43% of all quantified BLA-CeA projectors expressed CRHR1 in males and 15.11% expressed CRHR1 in females, and 6.28% of all quantified BLA-NAc projectors expressed CRHR1 in males and 6.47% expressed CRHR1 in females.

Together, this reveals that CRHR1 neurons in the BLA project to the NAc and CeA in both males and females, but with a greater proportion of CeA projectors expressing CRHR1. Interestingly, the BLA-NAc population may disproportionately possess more CRHR1+ neurons in females.

## 4 Discussion

Although CRHR1 signaling is involved in the stress response, its anatomical specificity has previously been difficult to characterize at a high resolution (Refojo et al., 2011). Here, we employed a recently developed transgenic CRHR1-Cre-tdTomato rat (Weera et al., 2022) to topographically map the distribution of BLA^CRHR1^ neurons and investigate their circuit identity in adult male and female rats. As a result, we established four central findings: (1) within the BLA, CRHR1 was expressed most strongly in ventral regions of the lateral amygdala and in caudal regions; (2) acute restraint stress increased FOS expression in BLA^CRHR1^ neurons, and stress-induced activation was strongest in the medial aspects of the BLA; (3) acute restraint stress significantly increased FOS expression on BLA-NAc, but not BLA-CeA projectors; and (4) CRHR1 neurons were expressed on a subset of BLA-CeA and BLA-NAc projection neurons. Together, our findings combine circuit- and molecular-specific approaches to provide insight into discrete BLA projection neuron populations activated by acute stress in adult male and female rats.

### 4.1 Topographical distribution and stress-induced activation of CRHR1 neurons in the BLA

Our topographical findings demonstrated a heterogenous pattern of CRHR1 expression in the BLA. The vast majority of CRHR1+ cells were expressed in the caudal regions of the BLA (AP -3.14 to AP -3.60) and particularly in the LA subregion. Although the LA is larger than other BLA subregions, we observed both a significantly greater percentage of total CRHR1+ cells in the LA as well as a greater density and can therefore be confident on a topographical bias. This bias towards the LA subregion strongly parallels findings demonstrated in transgenic CRHR1 reporter mice (Agoglia et al., 2020; Justice et al., 2008; Kühne et al., 2012) and agrees with evidence of the functional role of CRHR1 within the BLA. The majority of sensory input arrives into the amygdala via the LA (Sah et al., 2003), and this subregion is highly involved in learning tasks (Blair et al., 2001; Maren and Quirk, 2004). Thus, high expression of CRHR1 in the LA may allow for CRH release to mediate the “gain” of salient stimuli to facilitate learning, particularly during stress. Indeed, CRH delivered directly into the LA enhances excitability of neurons to afferent signals (Rainnie et al., 1992; Ugolini et al., 2008), and intra-BLA CRHR1 antagonists impair consolidation of aversive contextual memory (Hubbard et al., 2007; Roozendaal et al., 2002). Further, CRH directly modulates excitability of discrete circuits (Orozco-Cabal et al., 2008), and there is wide evidence that CRHR1 within the BLA interacts with other signaling molecules such as endocannabinoids (Gray et al., 2015, 2016), norepinephrine (Atsak et al., 2015), and glucocorticoids (Roozendaal et al., 2008). This collectively suggests that CRHR1 signaling may bias or enhance activation towards discrete circuits or cell types. As such, the role of CRHR1 signaling during stress may act to coordinate or enhance activation within the BLA.

Our mapping also revealed a caudal bias of CRHR1 expression in the BLA. This was surprising, as others have shown a caudal bias in BLA responsivity to rewarding stimuli (Kim et al., 2016). However, a rostral-caudal bias in response to valenced stimuli has not been consistently observed (Aukema et al., 2024; Beyeler et al., 2018; Piantadosi et al., 2023), suggesting complexity of activation biases within the BLA; for instance, CRHR1+ neurons may be a particular subset of caudal neurons that preferentially respond to aversive states. Conversely, a large proportion of caudal CRHR1+ neurons may instead be involved in reward processing and not be activated by stress at all. Indeed, we only observed a small proportion of all CRHR1+ neurons (<5%) that expressed FOS following stress, and this may represent only a subpopulation of CRHR1+ neurons that are active only in aversive states. Supporting this, CRHR1 signaling has also been implicated during reward, as CRHR1 signaling in the BLA has a role in regulating the strength of cocaine memory (Ritchie et al., 2021), and comparable levels of CRH release has been observed in the CeA following food intake as well as following restraint stress (Merali et al., 1998). Finally, an alternative role of CRHR1 may simply contribute to a general state of “salience” and thus be important in both aversive and appetitive states. As such, it remains essential to determine if BLA^CRHR1^ neurons are also activated during exposure to rewarding stimuli, and if activation occurs on the same neurons or even anatomical regions or circuits as those activated during stress.

Our findings demonstrate that CRHR1+ neurons exhibit increased FOS expression following exposure to stress, agreeing with previous work demonstrating that CRH is released during restraint stress and leads to activation of BLA projection neurons (Merlo Pich et al., 1995; Rostkowski et al., 2013). This specifically demonstrates that this effect is occurring on neurons expressing CRHR1, as CRHR2 is also known to be moderately expressed in the BLA (Van Pett et al., 2000). Although not surprising given the known role of CRHR1 in stress and anxiety-like behaviour, our findings are important as there is little understanding on the molecular identity of stress-responsive cells in the BLA. Apart from emerging work that has identified unique BLA projection populations, including those expressing *Ppp1r1b* (Kim et al., 2016; Zhang et al., 2020) or *Thy1* (Jasnow et al., 2013; McCullough et al., 2016) as fear-inhibiting, and projection populations expressing *Rspo2* (Kim et al., 2016), *CCK* (Shen et al., 2019), or *Crh* (Birnie et al., 2023) as promoting avoidance behaviours, there are few molecular markers to date for BLA neurons involved in aversive or rewarding states. This remains an important question for the field.

A further important question includes the source of source of CRH input within the BLA, as this remains unclear and largely speculative. CRH-releasing neurons are present within the BLA (Birnie et al., 2023; Sakanaka et al., 1987), perhaps allowing for local release. Alternatively, CRH may also diffuse from peptidergic neurons within the CeA (Bittencourt and Sawchenko, 2000; Roozendaal et al., 2002). Finally, CRH may be released from long-range inputs arising from the dorsal raphe or medial prefrontal cortex (Ritchie et al., 2024), although the evidence on each is so far limited.

### 4.2 Topographical distribution and stress-induced activation of BLA projection populations

Our findings support evidence from mice that the BLA-CeA and BLA-NAc projection populations are topographically distinct populations, with BLA-CeA projectors expressed laterally and BLA-NAc projectors expressed medially (Beyeler et al., 2018). We also observed a divergence in stress-induced activation, with BLA-NAc projectors, but not BLA-CeA projectors, activated by stress. These findings were surprising, given the canonical understanding of BLA-CeA neurons mediating avoidance, or “negative valence” behaviours (Beyeler et al., 2018; Kim et al., 2016), and BLA-NAc neurons mediating approach, or “positive valence” behaviours (Bagot et al., 2015; Beyeler et al., 2018; Kim et al., 2016; Namburi et al., 2015; Stuber et al., 2011). In contrast, our findings suggest that BLA-NAc neurons are more readily activated by acute stress than BLA-CeA neurons.

There are several possible explanations for why we may have observed minimal stress-induced activation in BLA-CeA projectors despite this overall population being intricately involved with aversive states. First, the BLA-CeA circuit is a large and highly heterogenous population, capable of both driving or inhibiting avoidance behaviours depending on precise cell type or microcircuit being activated (Ciocchi et al., 2010; Kim et al., 2017; Tye et al., 2011; Whittle et al., 2021). CTB-488 non-discriminably was expressed in a broad population of BLA-CeA projectors; as such, differences in stress-induced activation may be evident only if expression was restricted to a smaller, more specific subpopulation. Along the same lines, we quantified BLA-CeA projectors in two select regions-of-interest, only capturing cells within the junction between the basal amygdala and the ventrolateral LA or ventromedial LA. Although this region exhibits a large proportion of all BLA-CeA projectors, a notable population of BLA-CeA projectors also exists in the dorsal LA (Beyeler et al., 2018). Dorsal populations may display differences in excitability, including to aversive or valenced stimuli (Beyeler et al., 2018; Piantadosi et al., 2023). Notably, the LA receives dense sensory input from all sensory modalities (Sah et al., 2003), is activated by diverse novel and stressful stimuli (Aukema et al., 2024), and sends direct projections to the CeA which strongly undergo plasticity during fear learning (Li et al., 2013; Maren and Quirk, 2004). As such, this population of BLA-CeA projectors may be involved in mediating a “gain” on the stress signal, particularly in the context of fear or repeated stress. Given the presence of CRHR1 in the dorsal LA, and the importance of both the LA and CRHR1 in stress and fear (Gray et al., 2015; Roozendaal et al., 2002), differences in discrete anatomical or molecular subpopulations of BLA-CeA projectors remains an important question. Finally, while most experiments investigating valence-specific activation patterns involve repeated exposure to specific stimuli, we investigated activation patterns in response to a single, novel stressor. As such, BLA-CeA projectors may have a minimal impact during initial exposure to stress, and instead may play a more pronounced role following repeated exposures to an aversive stimulus. This is especially notable given the critical role of the BLA-CeA circuit in fear conditioning (Jimenez and Maren, 2009). It will be important to investigate how activation patterns within the BLA change with repeated exposure to stress and throughout learning.

In contrast to BLA-CeA projectors, we observed robust activation of BLA-NAc projectors following stress exposure. This was surprising given wide evidence for the BLA-NAc circuit in “positive valence” behaviours (Bagot et al., 2015; Beyeler et al., 2018; Kim et al., 2016; Namburi et al., 2015; Stuber et al., 2011). Like the BLA-CeA circuit, however, BLA-NAc projectors are a large and highly heterogenous population. In fact, emerging evidence has implicated subpopulations of BLA-NAc projectors driving aversive states (Birnie et al., 2023; Folkes et al., 2020; Shen et al., 2019). Alternatively, the BLA-NAc circuit has also been shown to mediate glucocorticoid-enhancement of memory (Roozendaal et al., 2001), suggesting a role in salience encoding. As such, it may be possible that this circuit is activated during both aversive and appetitive states to encode salience and drive arousal. It will thus be essential to investigate whether the same or different subpopulations of BLA-NAc neurons are activated during appetitive states, and whether magnitude to activation correlates with intensity of the stimulus.

### 4.3 BLA lateralization

Growing evidence suggests laterality of the amygdala, with the right amygdala particularly sensitive to pain (Allen et al., 2021) and controlling the expression of fear (Baker and Kim, 2004; Scicli et al., 2004). Hemispheric differences are also present with specific cell types, including lower expression of parvalbumin neurons in the right BLA (Butler et al., 2018). Further, amygdala lateralization can be differentially impacted by early life stress, which is also dependent on sex (Guadagno et al., 2020). We did not observe any significant differences between left and right BLA in either CRHR1 expression or stress-induced FOS expression, although there was a trend towards greater stress-induced FOS expression in the right hemisphere, particularly in the medial subregion of the BLA. Given that all CTB injections occurred in the right hemisphere, we are unable to make any conclusions on laterality of BLA-NAc or BLA-CeA projectors. However, we observed only marginally lower FOS expression within the left BLA, suggesting that both hemispheres are highly responsive to stress. Additionally, only bilateral disconnection of the BLA and CeA is sufficient to abolish expression of fear, while unilateral lesion of BLA-CeA connectivity in either hemisphere is insufficient (Jimenez and Maren, 2009). This suggests a conserved role of discrete circuits across hemispheres, at least in the context of fear. Collectively, these findings agree with prior literature that although both hemispheres are responsive to stress, the right amygdala may display greater sensitivity. As a result, lateralization of amygdala circuitry warrants further investigation.

### 4.4 Sex differences in BLA circuitry

Overall, we did not observe any major differences between males in females in overall circuitry and stress-induced activation within the BLA. We observed similar overall number and topography of CRHR1 neurons within the BLA, and both sexes exhibited similar topography of BLA-CeA and BLA-NAc projectors. Functionally, stress activated BLA^CRHR1^ neurons and BLA-NAc projectors, but not BLA-CeA projectors, in both males and females. This suggests that stress circuitry within the BLA is largely similar between sexes. We did, however, observe several subtle differences between sexes, particularly within the BLA-NAc population. Specifically, we observed a greater density of BLA-NAc projectors in females, and a greater proportion of stress-activated BLA-NAc projectors in males. Given the lower density of BLA-NAc projectors in males, a greater proportion of stress-activated BLA-NAc projectors may still represent a similar overall number of BLA-NAc projectors. Indeed, we observed no sex differences in number of BLA-NAc projectors activated by stress. Together, this suggests that although the BLA-NAc population may be denser in females, the number of neurons activated during stress is similar. As such, the additional number of BLA-NAc projectors in females may have a greater contribution in other states outside of stress.

These findings support growing evidence for subtle sexual dimorphism in BLA circuitry. For instance, a greater density of ventral hippocampus inputs is observed in the BLA of females than males (Huckleberry et al., 2023), and BLA-BST neurons exhibit lower excitability in females (Vantrease et al., 2022). In the context of NAc circuitry specifically, sex differences in synaptic connectivity have also been identified, with greater density and size of dendritic spines of NAc neurons (Forlano and Woolley, 2010). This may have particular consequence with respects to CRHR1 signaling, particularly during reward; indeed, CRHR1 signaling has sexually dimorphic effects in cocaine-memory learning, with stronger effects observed in females (Ritchie et al., 2021). Collectively, this suggests that the BLA^CRHR1^-NAc circuit may be especially impactful in females during learning.

### 4.5 Circuit identity of CRHR1 neurons in the BLA

Finally, we also investigated the circuit identity of BLA^CRHR1^ neurons. Both BLA-CeA and BLA-NAc projectors expressed CRHR1, suggesting that CRHR1 signaling within the BLA may act coordinatively to collectively activate cells from different projection neuron populations. We did, however, observe subtle differences in proportion of each projection neuron population expressing CRHR1. Although a relatively similar proportion of CRHR1 neurons projected to the CeA and NAc in males, there was a significantly greater proportion of CRHR1 neurons in females that projected to the NAc than the CeA. This agrees with the above discussion suggesting that the BLA^CRHR1^-NAc circuit may be especially important in females. Additionally, in both sexes, we did observe a greater proportion of BLA-CeA projectors that expressed CRHR1 than BLA-NAc projectors. This suggests that this projection population may be especially sensitive to the effects of CRHR1 signaling, and agrees with the importance of the BLA-CeA projection in expression of learned fear (Jimenez and Maren, 2009) and the role of BLA^CRHR1^ in aversive learning (Roozendaal et al., 2002, 2008).

In summary, we have demonstrated that CRHR1 is topographically distributed in the BLA in male and female rats, with greatest expression caudally and in the LA, and that BLA^CRHR1^ neurons are activated by acute stress. We have also identified that although BLA-NAc and BLA-CeA projectors both express CRHR1, these are topographically distinct and sexually dimorphic projection neuron populations that are differentially activated by acute stress. Collectively, this improves our understanding of the anatomical organization of the BLA and its functional contribution to stress by providing insight into the circuit-specific and molecular-specific identity of stress-responsive neurons in the BLA.

## CRediT authorship contribution statement

**Robert J. Aukema:** Writing – review & editing, Writing – original draft, Methodology, Investigation, Formal analysis, Conceptualization. **Gavin N. Petrie:** Writing – review & editing, Investigation. **Samantha L. Baglot:** Writing – review & editing, Investigation. **Nicholas W. Gilpin:** Writing – review & editing, Resources. **Matthew N. Hill:** Writing – review & editing, Writing – original draft, Supervision, Resources, Project administration, Methodology, Investigation, Funding acquisition, Formal analysis, Conceptualization.

## Data availability

Data will be made available on request.

## Funding

This research was supported by operating funds to MNH from the Canadian Institutes of Health Research (CIHR). RJA received salary support from the Mathison Centre for Mental Health Research & Education and the Cumming School of Medicine. GNP received salary support from BranchOut Neurological Foundation, CIHR, and the Cumming School of Medicine. SB received salary support from a Vanier Scholarship from CIHR.

## Declaration of competing interest

Authors have no conflicts of interests to report.

## Appendix A Supplementary data

Multimedia component 1 Multimedia component 1

Fig. S1 (A). Average width (um) of the BLA at each anterior-posterior plane (distance from bregma). There was a main effect of plane (F(6,56) = 55.20, p < 0.0001) but no effect of sex (F(1,56) = 1.451, p = 0.2335) or interaction (F(6,56) = 0.2810, p = 0.9436). (B) Average height (um) of the BLA at each anterior-posterior plane (distance from bregma). There was a main effect of plane (F(6,56) = 29.02, p < 0.0001) but no effect of sex (F(1,56) = 1.375, p = 0.2459) or interaction (F(6,56) = 0.4704, p = 0.8273). (C) Average triangular area (mm2) of the BLA at each anterior-posterior plane (distance from bregma). There was a main effect of plane (F(6,56) = 52.55, p < 0.0001) but no effect of sex (F(1,56) = 0.0073, p = 0.9324) or interaction (F(6,56) = 0.4659, p = 0.8306). Data were analyzed using an ordinary 2WAY ANOVA. Error bands represent mean+/-SEM. ∗p < 0.05, ∗∗p < 0.01, ∗∗∗p < 0.001, ∗∗∗∗p < 0.0001. Fig. S2 (A) There were no main effects of sex (F(1,76) = 0.0083, *p* = 0.9278), hemisphere (F(1,73) = 1.206, *p* = 0.2758), or subregion (F(1,76) = 1.183, *p* = 0.2802) on average number of CRHR1+ cells per image. Data were analyzed using a three-way ANOVA with data matched by hemisphere for each subregion and separated by sex (n = 18 males, n = 22 females) (B) There were no main effects of condition (F(1,38) = 0.3093, *p* = 0.5813) or subregion (F(1,38) = 2.725, *p* = 0.1070) on average number of CRHR1+ cells per image, or an interaction between the two (F(1,38) = 1.564, *p* = 0.2817). Data were analyzed using a 2Way RM ANOVA and include both sexes (naïve: n = 8 males, 13 females; stress: n = 10 males, 9 females) (C) There was significantly greater FOS in the medial BLA than the lateral BLA in animals exposed to stress (F(1,34) = 7.067, *p* = 0.0119), and no differences between sex (F(1,34) = 0.6003, *p* = 0.4438). There was a trend towards greater FOS expression in the right hemisphere (F((1,33) = 3.023, *p* = 0.0914) and a significant interaction between hemisphere and subregion (F(1,33) = 4.367, *p* = 0.0387), although no post-hoc differences were detected using Sidak's multiple comparisons test. Animals were analyzed from the stress condition only using a three-way ANOVA with data matched by hemisphere for each subregion and separated by sex (n = 10 males, 9 females). (D) Average number of BLA-NAc neurons also expressing FOS in the stress condition. Data were analyzed using an unpaired *t*-test (t(8) = 0.2785, *p* = 0.7877). Error bands represent mean+/-SEM. #*p* = 0.0846, ∗*p* < 0.05 Fig. S1
