# Supplementary material for: Acute stress activates basolateral amygdala neurons expressing corticotropin-releasing hormone receptor type 1 (CRHR1): Topographical distribution and projection-specific activation in male and female rats
Source: Neurobiol Stress. 2024 Nov 15;33:100694. doi: 10.1016/j.ynstr.2024.100694 (PMC11615582; doi:10.1016/j.ynstr.2024.100694)
Supplement: Multimedia component 1 [file mmc1.docx]

**SUPPLEMENTARY DATA**

**MATERIALS AND METHODS**

*Topographical mapping: Normalization.* For each section, DAPI staining (Sigma-Aldrich) was used to visually identify the shape of the BLA based on contours provided by the surrounding fiber tracts. We first established the average width and height of the BLA at 7 anterior-posterior (AP) positions. For every image, we identified its approximate AP position according to the Paxinos & Watson atlas (-2.12, -2.30, -2.56, -2.80, -3.14, -3.30, -3.60; (Paxinos & Watson, 2007)) and measured the average height, width, and triangular area of the BLA (**Figure 1B**) using Imaris Cell Imaging Software (Oxford Instruments). The formula for triangular area was: (height x width) / 2. Any image with a triangular area exceeding two times the standard deviation of the mean for the chosen AP position was re-assigned to a more appropriate AP position. We then calculated the average width (medial-lateral axis) and height (dorsal-ventral axis) at each of these 7 AP positions **(Supplementary Figure 1A-C)**, which was then rounded to the nearest 25um for standardization **(Supplementary Table 1)**.

Next, we normalized coordinates of every RFP+ neurons from each image according to these standardized dimensions. For each raw coordinate, the x-coordinate was normalized to the average width of the BLA at that AP position, and the y-coordinate to the average height of the BLA at that AP position. This established new x,y coordinates that maintained their original relative position in the BLA but could now be directly compared to images with a BLA of different raw dimensions.

To subdivide the BLA into the LA, LBA, and mBA subdivisions and to account for the curvature of the BLA along the medial fiber tract, a template shape was first created by manually fitting a standardized shape of the BLA, including each subregion, derived from the Paxinos & Watson atlas (Paxinos & Watson, 2007) to all normalized RFP+ neurons in each plane and then excluding FRP+ points outside of this template. In total, the standardized shape fit 93.39% (8,302 of 8,890) of labelled cells. This established a standardized template comprised of 25um x 25um “pixels” used for further representation and quantification (see **Figure 1C** for example).

*Topographical mapping: Visualization.* To visually represent density gradients across the BLA after normalization, the average density of RFP+ neurons were calculated per 25um x 25um bin and represented in heatmaps using a custom MATLAB script. Each pixel value represents average density per image, averaged across animals in the same group.

*Topographical mapping: Quantification.* We calculated RFP+ density in each subregion for each animal individually and then compared group means. The total area of a subregion was calculated as: [(number of 25umx25um pixels comprising the subregion of interest) * 25um * 25um]. The density was then calculated as: [total # of RFP+ cells detected in all pixels comprising the subregion of interest / total area of subregion of interest]. As multiple images were often collected from each AP plane for each animal, data were analyzed as average density per image: [(total # of cells from all pixels comprising the subregion of interest, from all slices) / (total area of subregion of interest * number of slices)]. These calculations were streamlined using a custom MATLAB script that can be accessed at the authors’ request ([raukema@mclean.harvard.edu](mailto:raukema@mclean.harvard.edu) or [mnhill@ucalgary.ca](mailto:mnhill@ucalgary.ca)).

**Supplementary Table 1.** Standardized width (medial-lateral axis) and height (dorsal-ventral axis) at each AP position

| **AP** | **Width (um)** | **Height (um)** |
| --- | --- | --- |
| -2.12 | 600 | 1450 |
| -2.30 | 700 | 1650 |
| -2.56 | 850 | 1950 |
| -2.80 | 1075 | 2000 |
| -3.14 | 1125 | 2175 |
| -3.30 | 1275 | 2400 |
| -3.60 | 1300 | 2450 |
