# Supplementary figures and images for: Acute stress activates basolateral amygdala neurons expressing corticotropin-releasing hormone receptor type 1 (CRHR1): Topographical distribution and projection-specific activation in male and female rats
Source: Neurobiol Stress. 2024 Nov 15;33:100694. doi: 10.1016/j.ynstr.2024.100694 (PMC11615582; doi:10.1016/j.ynstr.2024.100694)

Supplementary Figure 1

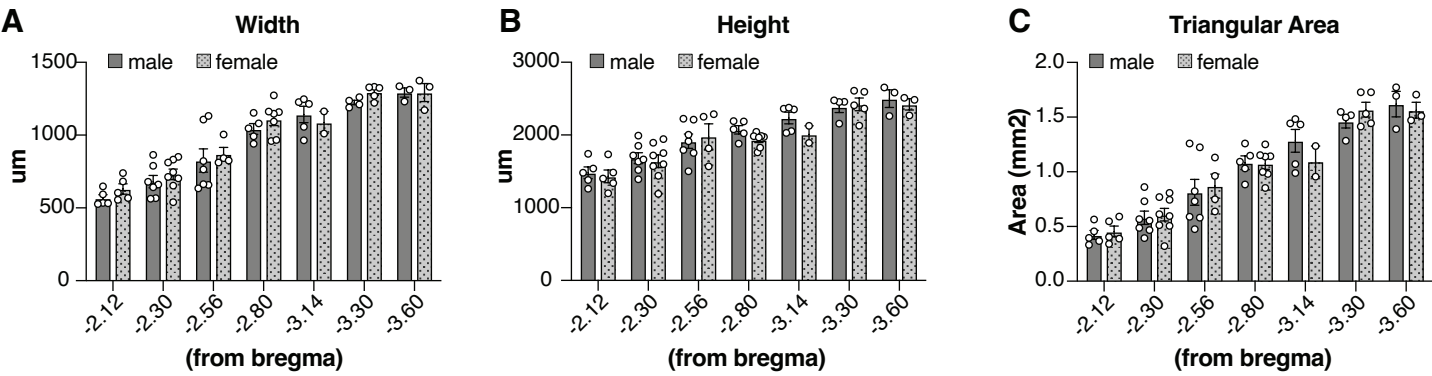

Supplementary Figure 2

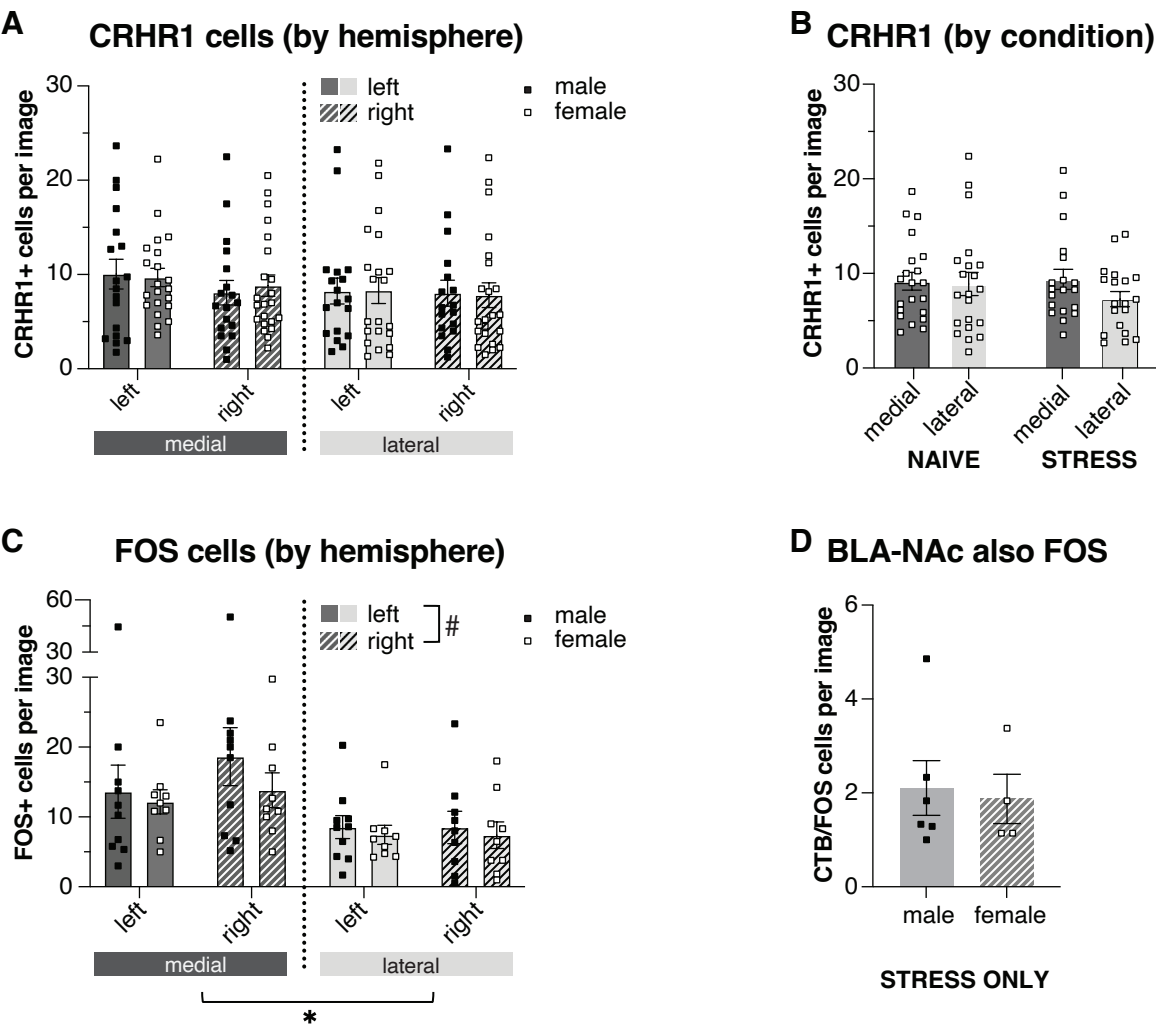

Supplement: Fig. S1 — (A). Average width (um) of the BLA at each anterior-posterior plane (distance from bregma). There was a main effect of plane (F(6,56) = 55.20, p < 0.0001) but no effect of sex (F(1,56) = 1.451, p = 0.2335) or interaction (F(6,56) = 0.2810, p = 0.9436). (B) Average height (um) of the BLA at each anterior-posterior plane (distance from bregma). There was a main effect of plane (F(6,56) = 29.02, p < 0.0001) but no effect of sex (F(1,56) = 1.375, p = 0.2459) or interaction (F(6,56) = 0.4704, p = 0.8273). (C) Average triangular area (mm2) of the BLA at each anterior-posterior plane (distance from bregma). There was a main effect of plane (F(6,56) = 52.55, p < 0.0001) but no effect of sex (F(1,56) = 0.0073, p = 0.9324) or interaction (F(6,56) = 0.4659, p = 0.8306). Data were analyzed using an ordinary 2WAY ANOVA. Error bands represent mean+/-SEM. ∗p < 0.05, ∗∗p < 0.01, ∗∗∗p < 0.001, ∗∗∗∗p < 0.0001. Fig. S2 (A) There were no main effects of sex (F(1,76) = 0.0083, p = 0.9278), hemisphere (F(1,73) = 1.206, p = 0.2758), or subregion (F(1,76) = 1.183, p = 0.2802) on average number of CRHR1+ cells per image. Data were analyzed using a three-way ANOVA with data matched by hemisphere for each subregion and separated by sex (n = 18 males, n = 22 females) (B) There were no main effects of condition (F(1,38) = 0.3093, p = 0.5813) or subregion (F(1,38) = 2.725, p = 0.1070) on average number of CRHR1+ cells per image, or an interaction between the two (F(1,38) = 1.564, p = 0.2817). Data were analyzed using a 2Way RM ANOVA and include both sexes (naïve: n = 8 males, 13 females; stress: n = 10 males, 9 females) (C) There was significantly greater FOS in the medial BLA than the lateral BLA in animals exposed to stress (F(1,34) = 7.067, p = 0.0119), and no differences between sex (F(1,34) = 0.6003, p = 0.4438). There was a trend towards greater FOS expression in the right hemisphere (F((1,33) = 3.023, p = 0.0914) and a significant interaction between hemisphere and subregion (F(1,33) = 4. [file mmc2.pdf]
